# Survival Disparities in Adenocarcinoma Within Adenomatous Polyps: A National Cancer Database Analysis

**DOI:** 10.7759/cureus.84792

**Published:** 2025-05-25

**Authors:** Catherine S Taghizadeh, Beau Hsia, Yanick Tade, Susan Rafie, Akaash Surendra, Peter T Silberstein, Deepak Vadehra, Timothy J Brown

**Affiliations:** 1 Department of Medicine, Creighton University School of Medicine, Phoenix, USA; 2 Department of Oncology, Creighton University School of Medicine, Omaha, USA; 3 Department of Osteopathic Medicine, Midwestern University Arizona College of Osteopathic Medicine, Glendale, USA; 4 Department of Oncology, Arizona State University, Tempe, USA; 5 Department of Medical Oncology, Roswell Park Comprehensive Cancer Center, Buffalo, USA; 6 Department of Internal Medicine, Abramson Cancer Center, University of Pennsylvania, Philadelphia, USA

**Keywords:** adenocarcinoma in adenomatous polyp, national cancer database and seer analyses, ncdb, prognostic factors, socioeconomic factors

## Abstract

Purpose

Adenocarcinoma within adenomatous polyps is a rare malignancy characterized by outward growths from the lining of the colon or rectum. These neoplasms can develop over several years, with only a small proportion of adenomatous polyps demonstrating malignant potential. Information and treatment options are limited due to the rarity of this cancer. This retrospective cohort study aims to identify and analyze demographic and prognostic factors associated with overall survival and mortality in patients diagnosed with adenocarcinoma within adenomatous polyps.

Methods

Patients diagnosed with adenocarcinoma within adenomatous polyps between 2004 and 2020 were identified using the histology code 8210 through the National Cancer Database (NCDB). Kaplan-Meier curves, multivariable Cox proportional hazards regression analyses, and locally estimated scatterplot smoothing (LOESS) were utilized to assess whether there are statistically significant relationships between demographic and prognostic factors and survival in patients diagnosed with adenocarcinoma within adenomatous polyps.

Results

A cohort of 58,643 patients with adenocarcinoma within adenomatous polyps was analyzed to identify factors influencing survival. Older age was associated with poorer overall survival, with 5-year and 10-year survival rates of 52% (n = 7,864) and 25% (n = 3,750), respectively, in patients aged 76-100 years. Females exhibited better survival outcomes, with 5-year and 10-year survival rates of 74% (n = 20,439) and 57% (n = 15,626), compared to males (5-year: 71%, n = 22,031; 10-year: 53%, n = 16,485). The Black race had a 69% increased mortality risk compared to other races (hazard ratio (HR) = 0.69; 95% confidence interval (CI): 0.64-0.75, p < 0.001). Patients with a Charlson-Deyo (CD) comorbidity score of 1 (HR = 1.30; 95% CI: 1.26-1.34, p < 0.001) and 2 (HR = 1.69; 95% CI: 1.62-1.77, p < 0.001) had higher mortality risks, with a 30% and 69% increased risk, respectively, compared to those with a score of 0. Medicare (HR = 1.25; 95% CI: 1.20-1.29, p < 0.001) and Medicaid (HR = 1.67; 95% CI: 1.55-1.79, p < 0.001) insurance were also associated with decreased survival. Survival probability declined with advanced cancer stage, with Stage IV patients experiencing a 10-fold increased mortality risk compared to Stage I (HR = 10.41; 95% CI: 9.91-10.94, p < 0.001).

Conclusions

Older age, male sex, Black race, Medicare/Medicaid insurance, higher CD score, advanced cancer stage, lower education status, and lower income were all associated with poorer survival outcomes.

## Introduction

Colorectal cancer (CRC) is a leading cause of cancer-related mortality in the United States [1-4]. Adenocarcinoma arising within adenomatous polyps, a specific CRC subtype, originates from exophytic growths in the colonic or rectal mucosa. The adenoma-carcinoma sequence is the well-established pathway for the development of the majority of CRCs, involving the progression from benign adenomatous polyps to invasive adenocarcinoma over time. While this transformation is a common oncogenic process in CRC, the specific scenario of detecting and diagnosing an invasive adenocarcinoma predominantly confined within an adenomatous polyp at the time of initial pathological evaluation is relatively less frequent in the broader context of established CRC. These lesions often have the potential for detection at earlier stages during routine endoscopic surveillance, which may influence the spectrum of prognostic factors compared to CRCs diagnosed at more advanced stages. While prognostic factors for general colon adenocarcinoma are well documented, data on this distinct entity remain scarce. The unique biological context of malignant transformation within a polyp, and the initial treatment via polypectomy may lead to different determinants of survival compared to other CRC presentations. While most colorectal polyps are benign, 5%-10% undergo malignant transformation over several years, influenced by factors such as polyp size and histologic subtype [1,5,6]. These malignancies often present with nonspecific symptoms, including rectal bleeding, diarrhea, constipation, and abdominal pain, mirroring the underlying polyp’s clinical manifestations [7]. While adenocarcinoma arising within adenomatous polyps constitutes a smaller proportion of all CRCs, it represents a critical clinical transition for a significant number of patients undergoing colonoscopic surveillance. Emerging research suggests potential biological distinctions in these polyp-associated cancers, including mutations in TP53, FBXW7, PIK3CA, KIAA1804, and SMAD2. Understanding the unique prognostic factors in this subtype is therefore crucial for optimizing management strategies and improving outcomes for these patients [8].

Treatment typically involves polypectomy and surgical resection, with adjuvant therapies such as radiation and chemotherapy tailored to patient demographics (age and sex), polyp characteristics (size and location), and comorbidities [6,9]. Younger patients often receive adjuvant therapies to optimize outcomes [10]. Despite advances in treatment, survival disparities persist: the National Cancer Institute's Surveillance, Epidemiology, and End Results (SEER) Program reports five-year relative survival rates of 63% for colon cancer and 68% for rectal cancer, with better outcomes in women and White patients, compared to men and Black patients [11,12].

Utilizing a retrospective cohort study design, we will analyze data from the National Cancer Database (NCDB) to identify key demographic and prognostic factors affecting survival outcomes in adenocarcinoma arising specifically within adenomatous polyps, a subtype underrepresented in existing literature. While numerous studies have investigated prognostic factors in general colorectal adenocarcinoma, large-scale analyses specifically examining adenocarcinoma arising within adenomatous polyps using national databases have been limited. By leveraging a large, population-based dataset, we aim to identify prognostic factors associated with overall survival, including age, sex, race, income, education, insurance status, tumor size, pathological stage, treatment modalities (radiation and chemotherapy), distance traveled for care, and Charlson-Deyo (CD) score, a measure of comorbidity burden.

Our analysis seeks to address critical gaps in understanding this rare malignancy, providing insights into the interplay of clinical and socioeconomic factors on survival outcomes. By elucidating these relationships, we aim to inform tailored treatment strategies and reduce disparities in care for patients with adenocarcinoma within adenomatous polyps.

## Materials and methods

This is a retrospective cohort study of patients within the NCDB diagnosed with adenocarcinoma within adenomatous polyps from 2004 to 2020 [13]. Selecting this 17-year window provided a robust sample size (n = 58,643) of patients with adenocarcinoma within adenomatous polyps, which is crucial for detecting statistically significant associations, given the rarity of this specific diagnosis. No specific reference number is assigned by the NCDB for data searches. The NCDB is a clinical oncology database derived from hospital registry data, with information on patient characteristics, tumor staging, tumor histology, type of first treatment, disease recurrence, and survival from more than 1,500 Commission on Cancer (CoC)-accredited facilities. This de-identified patient data was made accessible to the authors through the Participant User Data Files program [13].

Patients with adenocarcinoma within adenomatous polyps were identified from NCDB data using the ICD-O-3 (a coding system that lists tumor site, histology, and behavior) histology code 8210, which specifically classifies adenocarcinoma in adenomatous polyp tumors based on their morphological and histological features. Patients were then selected if they had a behavior code 3, indicating an invasive characteristic. Patients were excluded from the cohort if they had any missing clinical or demographic factors, or if they had concurrent tumors. Patients with missing data for key demographic or clinical variables were excluded to ensure the integrity and completeness of the dataset for the multivariable analyses. Imputation methods were not employed due to the potential for introducing further uncertainty in a dataset of this size and complexity.

Covariates

Patients were analyzed by age, sex, race, education, income, insurance status, tumor size, analytical stage (the extent of the cancer, including size and spread), CD score, primary anatomic site, primary radiation, primary and adjuvant chemotherapy, and distance traveled for health care. Race was categorized into three groups: White, Black, and Other. The race group categorized as Other includes American Indian, Aleutian or Eskimo, Chinese, Japanese, Filipino, Hawaiian, Korean, Vietnamese, Kampuchean, Asian Indian or Pakistani not otherwise specified (NOS), Asian Indian, Micronesian NOS, Other Asian, Asian NOS, Oriental NOS, and Pacific Islander NOS. Income was measured by median household income from 2016 to 2020 for the ZIP code where the patient resided at the time of diagnosis. Education was measured in 2020 by the percentage of residents within the patient’s ZIP code of residence who did not graduate from high school. The staging was measured by the NCDB analytical stage, a categorical variable (Stages I-IV). Clinical staging was used when the NCDB analytical stage variable was not available. Insurance status was categorized into five groups: uninsured, private, Medicare, Medicaid, and other government insurance. The primary anatomic site was divided into seven groups using ICD-O-3 topography codes: cecum (C180), ascending colon (C182), transverse colon (C184), sigmoid colon (C187), rectosigmoid junction (C199), rectum (C209), and other. Distance traveled for health care was measured by the miles between the patient’s residence and the hospital that reported the case. The CD score, which measures comorbidities, was used as a categorical variable to classify patients into groups with scores of 0, 1, 2, and ≥3.

The primary outcome of interest was overall survival, defined from the date of diagnosis until the date of death, censored at last contact in the database. Overall survival encompasses death from any cause, as recorded in the NCDB. Independent prognostic factors were identified using a multivariable Cox hazard regression model. Kaplan-Meier curves were generated to visualize survival differences, and overall survival probabilities at 2, 5, and 10 years were estimated from survival tables. Variables included in the multivariable Cox model were age, sex, race, insurance status, education, income, CD score, analytical stage, primary anatomic site, primary radiation, and primary and adjuvant chemotherapy, all of which were a priori variables of interest. Patients within the same facility were accounted for using a robust sandwich covariance matrix. LOESS (locally estimated scatterplot smoothing) methods were used to examine the functional form of continuous variables, while the log-negative-log survival curves and statistical interaction with time were used to evaluate the proportional hazards assumption for each variable. All variables met this assumption.

Statistical considerations

The descriptive statistics, unadjusted survival analysis, and multivariable analysis for this study were conducted using IBM SPSS Statistics for Windows, Version 27 (Released 2020; IBM Corp., Armonk, NY, USA). Patients with any missing clinical or demographic factors were excluded from the cohort. Bonferroni correction was used to adjust the p-value threshold for multiple comparisons, ensuring that the family-wise error rate remained controlled at α = 0.05.

Oversight

The University of Arizona Biomedical Institutional Review Board (IRB) reviewed this study (IRB Submission ID: STUDY00003534) and determined that it does not involve human subjects research, as defined by the Department of Health and Human Services (DHHS) and Food and Drug Administration (FDA) regulations. Consequently, IRB approval and ongoing review were not required.

## Results

Demographics

The NCDB includes a total of 17,886,685 patients from 2004 to 2020. Of these, 77,161 patients were found to have adenocarcinoma within adenomatous polyps, while 58,643 (76.0%) patients had no missing information. Patient demographics and clinical characteristics are summarized in Table 1. The cohort exhibited a slight male predominance (n = 30,986, or 53%) and was predominantly White (n = 49,407, or 84%). The median age at diagnosis was 67 years. Patients were distributed across income quartiles, with the highest proportion (n = 22,843, or 39%) in the highest income quartile (≥ $74,063). Similarly, educational attainment was distributed across quartiles, with the largest proportion (n = 17,675, or 30%) in the second-highest quartile. The majority of patients had Medicare insurance (n = 30,508, or 52%), followed by private insurance (n = 23,618, or 40%). The cohort was relatively healthy, with 70% having a CD score of 0 (n = 40,986). The most common primary tumor sites, encompassing 70% of the cohort, were the sigmoid colon (n = 14,691, or 25%), rectum (n = 11,660, or 20%), ascending colon (n = 7,613, or 13%), and cecum (n = 7,137, or 12%). The majority of patients underwent surgical resection (n = 52,647, or 90%), with a subset receiving primary or adjuvant therapies.

**Table 1:** Demographic and clinical characteristics of 58,643 patients with adenocarcinoma within adenomatous polyps NCDB Stage I: small, localized tumor; NCDB Stage II: larger localized tumor mass, with slight lymph node and tissue involvement; NCDB Stage III: large tumor, with regional lymph node and tissue involvement; NCDB Stage IV: metastatic cancer (cancer that has spread to distant parts of the body). NCDB, National Cancer Database; NOS, Not Otherwise Specified

Variable		N (%)
Sex	Male	30,986 (52.8)
	Female	27,657 (47.2)
Race	White	49,407 (84.3)
	Black	6,063 (10.3)
	Other	3,173 (5.4)
Age (years)	Mean ± Standard deviation	66.04 ± 12.80
	Median (interquartile range)	67 (19)
Zip code-level median household income (2016-2020, $)	< $46,277	9,072 (15.5)
	$46,277-$57,856	12,340 (21.0)
	$57,857-$74,062	14,388 (24.5)
	≥ $74,063	22,843 (39.0)
Zip code-level education (% without high-school degree, 2020)	≥ 15.3%	11,509 (19.6)
	9.1%-15.2%	15,887 (27.1)
	5%-9%	17,675 (30.1)
	< 5%	13,572 (23.1)
Insurance status	Uninsured	1,219 (2.1)
	Private	23,618 (40.3)
	Medicaid	2,747 (4.7)
	Medicare	30,508 (52.0)
	Other government	551 (0.9)
Distance traveled for healthcare (miles)	Mean ± Standard deviation	19.66 ± 59.51
	Median (interquartile range)	8.5 (14.8)
Charlson-Deyo comorbidity score	0	40,986 (69.9)
	1	12,105 (20.6)
	2	3,587 (6.1)
	≥ 3	1,965 (3.4)
Tumor size (mm)	Mean ± Standard deviation	29.94 ± 25.21
	Median (interquartile range)	25.00 (32.00)
NCDB analytical stage	I	37,867 (64.6)
	II	7,380 (12.6)
	III	9,413 (16.1)
	IV	3,983 (6.8)
Treatment	Received surgery	52,647 (89.8)
	Received primary chemotherapy	14,065 (24.0)
	Received primary radiation	6,345 (10.8)
Surgical margins	No residual tumor	51,642 (88.1)
	Residual tumor, NOS	679 (1.2)
	Microscopic residual tumor	1,115 (1.9)
	Macroscopic residual tumor	151 (0.3)

Age

**Figure 1:**
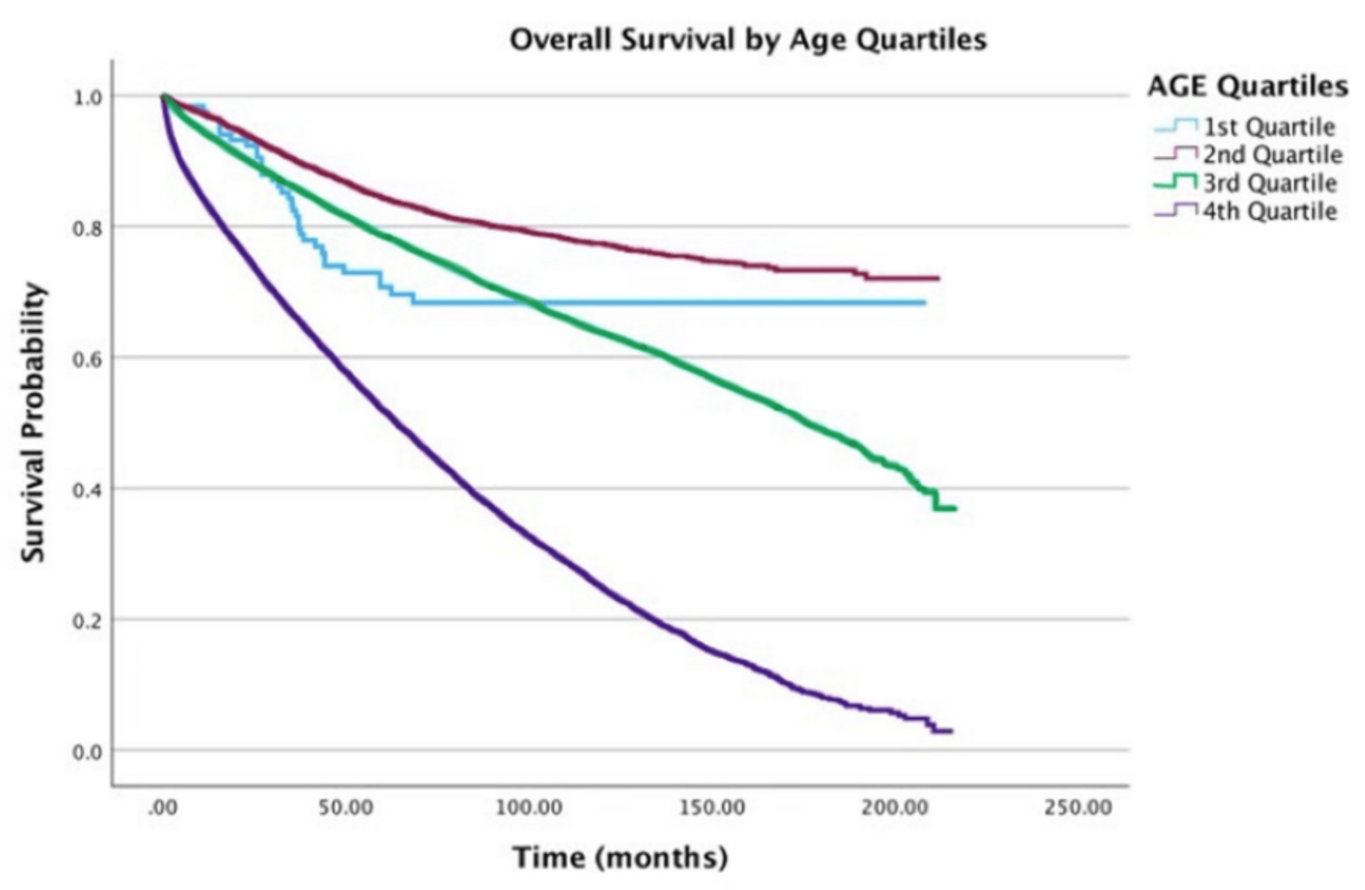
Overall survival by age of patients with adenocarcinoma within adenomatous polyps (N = 58,643) Age of patients is measured in quartiles: First quartile, 0-25 years; Second quartile, 26-50 years; Third quartile, 51-75 years; Fourth quartile, 76-100 years.

The following 2-, 5-, and 10-year survival estimates by variable are shown in Table 2. Table 3 shows the results of the multivariate Cox regression for survival analysis. Increasing age was associated with a higher hazard of death (Figure 1). Both 5- and 10-year survival rates declined progressively with age, with the oldest cohort (76-100 years) demonstrating the poorest outcomes (5-year: 52%; 10-year: 25%). Multivariable Cox regression revealed a 30% increase in the hazard of death for every five-year increment in age (hazard ratio (HR) = 1.30; 95% confidence interval (CI): 1.29-1.31, p < 0.001).

**Table 2:** Median 2-, 5-, and 10-year survival estimates of 58,643 patients with adenocarcinoma within adenomatous polyps NCDB, National Cancer Database

Variable		2-year, N (%)	5-year, N (%)	10-year, N (%)
Sex	Male	26,555 (85.7)	22,031 (71.1)	16,485 (53.2)
	Female	24,117 (87.2)	20,439 (73.9)	15,626 (56.5)
Race	White	42,737 (86.5)	35,771 (72.4)	26,828 (54.3)
	Black	5,050 (83.3)	4,190 (69.1)	3,201 (52.8)
	Other	2,865 (90.3)	2,523 (79.5)	2,151 (67.8)
Zip code-level median household income (2020, $)	< $46,277	7,503 (82.7)	6,133 (67.6)	4,445 (49.0)
	$46,227-$57,856	10,526 (85.3)	8,638 (70.0)	6,392 (51.8)
	$57,857-$74,062	12,417 (86.3)	10,316 (71.7)	7,683 (53.4)
	≥ $74,063	20,216 (88.5)	17,384 (76.1)	13,591 (59.5)
Zip code-level education (2020, % No high-school diploma)	≥ 15.3%	9,702 (84.3)	8,045 (69.9)	6,065 (52.7)
	9.1%-15.2%	13,599 (85.6)	11,264 (70.9)	8,404 (52.9)
	5.0%-9.0%	15,377 (87.0)	12,867 (72.8)	9,686 (54.8)
	< 5.0%	11,971 (88.2)	10,301 (75.9)	7,967 (58.7)
Age (years)	0-25	111 (92.3)	85 (70.8)	82 (68.4)
	26-50	6,319 (93.7)	5,685 (84.3)	5,219 (77.4)
	51-75	32,942 (89.9)	28,838 (78.7)	23,378 (63.8)
	76-100	11,283 (74.6)	7,864 (52.0)	3,750 (24.8)
NCDB analytical stage	I	34,535 (91.2)	30,256 (79.9)	23,288 (61.5)
	II	32,376 (85.5)	26,166 (69.1)	18,328 (48.4)
	III	8,010 (85.1)	6,410 (68.1)	4,810 (51.1)
	IV	1,804 (45.3)	693 (17.4)	362 (9.1)

**Table 3:** Multivariable Cox regression model of 58,643 patients with adenocarcinoma within adenomatous polyps Age (per five-year increase): hazard ratio reflects the change in hazard for each five-year increase in age.

Variable		HR (95% CI)	p-values
Age (per 5-year increase)		1.30 (1.29-1.31)	<0.001
Sex	Males vs. females	0.84 (0.82-0.86)	<0.001
Race and ethnicity	White vs. Black	1.07 (1.02-1.12)	0.002
	White vs. Other	0.74 (0.69-0.79)	<0.001
	Black vs. Other	0.69 (0.64-0.75)	<0.001
Charlson-Deyo score	0 vs. 1	1.30 (1.26-1.34)	<0.001
	0 vs. 2	1.69 (1.62-1.77)	<0.001
	0 vs. ≥ 3	2.32 (2.19-2.45)	<0.001
	1 vs. 2	1.31 (1.24-1.37)	<0.001
	1 vs. ≥ 3	1.79 (1.68-1.90)	<0.001
	2 vs. ≥ 3	1.37 (1.27-1.47)	<0.001
Zip code-level median household income (2020 US Dollars)	< $46,277 vs. $46,227-$57,856	0.95 (0.91-0.99)	0.025
	< $46,277 vs. $57,857-$74,062	0.92 (0.88-0.96)	<0.001
	< $46,277 vs. ≥ $74,063	0.82 (0.78-0.86)	<0.001
Zip code-level education (2020, % No high-school diploma)	≥ 15.3% vs. 9.1%-15.2%	1.00 (0.96-1.04)	0.961
	≥ 15.3% vs. 5.0%-9.0%	0.99 (0.95-1.03)	0.167
	≥ 15.3% vs. < 5.0%	0.93 (0.89-0.98)	0.004
Insurance	Private vs. None	1.56 (1.51-1.62)	<0.001
	Private vs. Medicaid	1.67 (1.55-1.79)	<0.001
	Private vs. Medicare	1.25 (1.20-1.29)	<0.001
	Private vs. Other government	1.02 (0.87-1.19)	0.834
Treatment	No primary radiation vs. Primary radiation	1.28 (1.22-1.34)	<0.001
	No primary chemotherapy vs. Primary chemotherapy	0.86 (0.81-0.91)	<0.001
	No adjuvant chemotherapy vs. Adjuvant chemotherapy	0.79 (0.74-0.83)	<0.001
Analytical stage	Stage I vs. Stage II	1.38 (1.33-1.44)	<0.001
	Stage I vs. Stage III	2.04 (1.95-2.13)	<0.001
	Stage I vs. Stage IV	10.41 (9.91-10.94)	<0.001
Primary anatomic site	Rectosigmoid junction vs. rectum	1.10 (1.03-1.17)	0.003
	Rectosigmoid junction vs. other	1.20 (1.13-1.28)	<0.001

Sex

Both unadjusted and adjusted survival analyses revealed increased survival rates associated with the female sex. The 5- and 10-year survival rates were marginally higher for females (74% and 57%, respectively) compared to males (71% and 53%, respectively). Multivariable Cox regression revealed that females maintained a statistically significant survival advantage, with a 16% lower hazard of death (HR = 0.84; 95% CI: 0.82-0.86, p < 0.001).

Race

**Figure 2:**
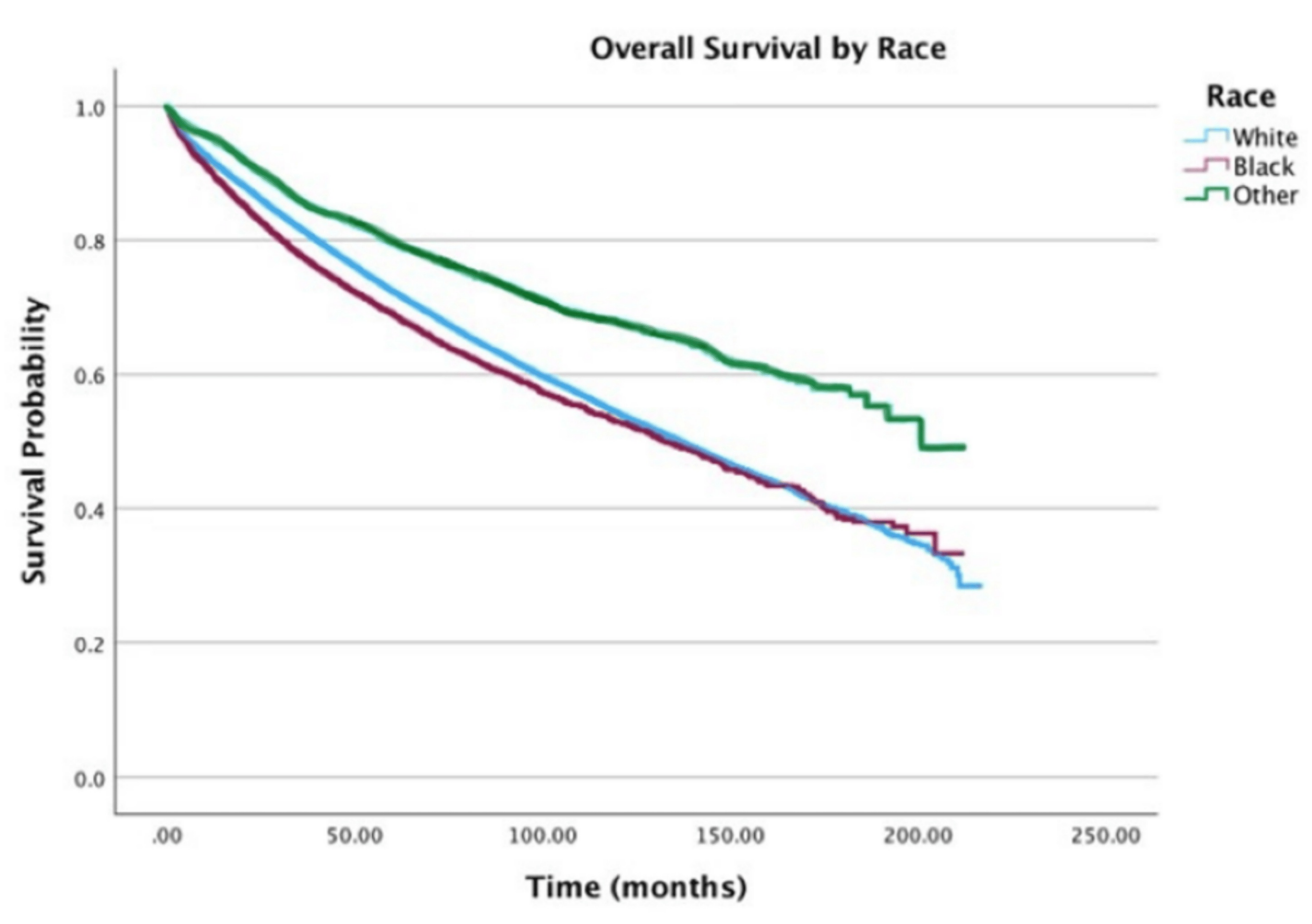
Overall survival by race of patients with adenocarcinoma within adenomatous polyps (N = 58,643)

Racial disparities persisted (Figure 2), with patients categorized as “Other” race exhibiting the highest five-year survival (80%), compared to 72% for White patients and 69% for Black patients. Adjusted analysis showed the “Other” race group had a 31% lower hazard of mortality relative to the Black race (HR = 0.69; 95% CI: 0.64-0.75, p < 0.001).

CD scores

Higher CD scores correlated with poorer survival, with scores of 1 (HR = 1.30; 95% CI: 1.26-1.34) and ≥2 (HR = 1.69; 95% CI: 1.62-1.77) associated with a 30% and 69% increased hazard of death, respectively (p < 0.001 for both).

Income

**Figure 3:**
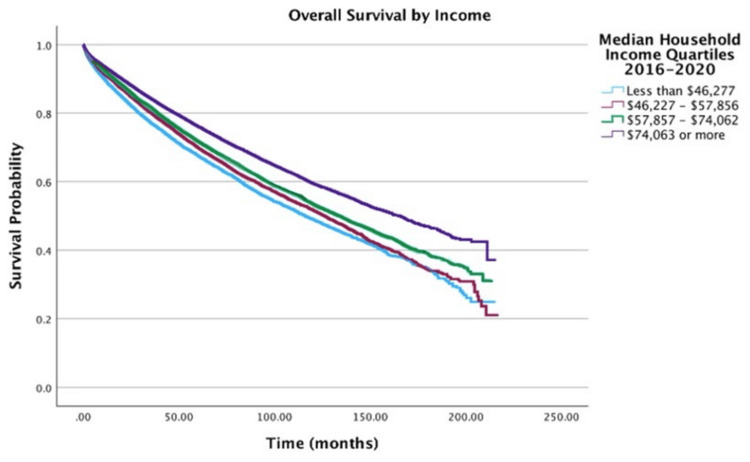
Overall survival by income of patients with adenocarcinoma within adenomatous polyps (N = 58,643)

Socioeconomic factors significantly influenced outcomes. Compared to the highest income quartile (≥ $74,063), all lower income brackets exhibited elevated hazards of death: <$46,277: HR = 1.22 (95% CI: 1.16-1.28, p < 0.001); $46,277-57,857: HR = 1.16 (95% CI: 1.11-1.22, p < 0.001); $57,857-74,063: HR = 1.11 (95% CI: 1.07-1.15, p < 0.001) (Figure 3).

Insurance

**Figure 4:**
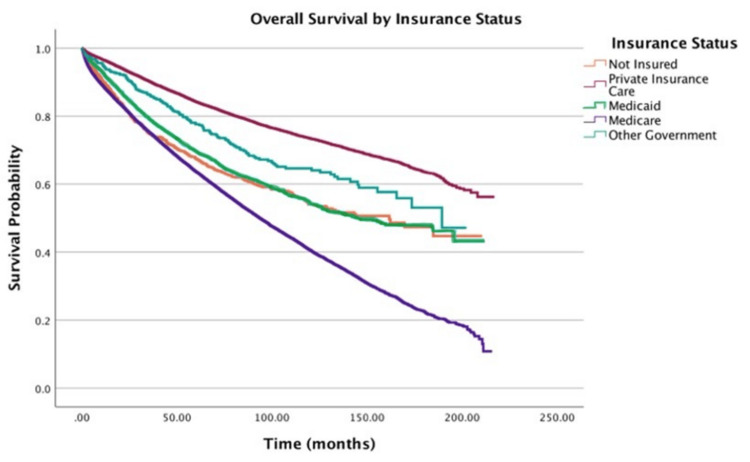
Overall survival by insurance status of patients with adenocarcinoma within adenomatous polyps (N = 58,643)

Insurance status further stratified survival: Medicare (HR = 1.25; 95% CI: 1.20-1.29) and Medicaid (HR = 1.67; 95% CI: 1.55-1.79) were associated with a 1.25- and 1.67-fold higher hazard of death, respectively, compared to private insurance (p < 0.001 for both). Medicaid patients also demonstrated a 33% higher hazard relative to Medicare recipients (HR = 1.33; 95% CI: 1.25-1.43, p < 0.001). Figure 4 shows overall survival by insurance status.

Primary site

**Figure 5:**
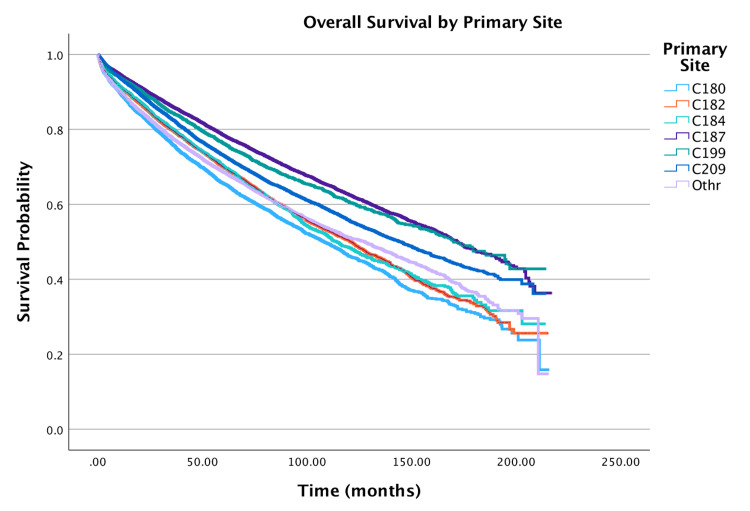
Overall survival by primary site of patients with adenocarcinoma within adenomatous polyps (N = 58,643) Primary site codes: C180, Cecum; C182, Ascending colon; C184, Transverse colon; C187, Sigmoid colon; C199, Rectosigmoid junction; C209, Rectum.

Overall survival by primary anatomic site of the tumor is shown in Figure 5. The primary tumor site significantly influenced survival outcomes. Compared to the reference category (rectosigmoid junction), the cecum (HR = 1.11; 95% CI: 1.06-1.17, p < 0.001), ascending colon (HR = 1.18; 95% CI: 1.13-1.24, p < 0.001), transverse colon (HR = 1.10; 95% CI: 1.04-1.16, p < 0.001), and sigmoid colon (HR = 1.22; 95% CI: 1.17-1.27, p < 0.001) were associated with a higher hazard of death. Similarly, rectal tumors exhibited a 9% increased hazard relative to the rectosigmoid junction (HR = 1.09; 95% CI: 1.04-1.14, p < 0.001).

Stage

The advanced disease stage correlated with progressively worse survival. Compared to Stage I, Stage II (HR = 1.38; 95% CI: 1.33-1.44, p < 0.001), Stage III (HR = 2.04; 95% CI: 1.95-2.13, p < 0.001), and Stage IV (HR = 10.41; 95% CI: 9.91-10.94, p < 0.001) demonstrated 38%, 104%, and 941% higher hazards of death, respectively. Correspondingly, 5- and 10-year survival probabilities declined sharply with advancing stage.

Distance travelled and education

No association was observed between the distance traveled for healthcare and survival. However, education level revealed disparities: the highest quartile (with the least number of individuals without a high school diploma) had reduced hazards compared to the second-highest (HR = 0.94; 95% CI: 0.90-0.97, p < 0.001), third-highest (HR = 0.93; 95% CI: 0.89-0.97, p < 0.001), and lowest quartiles (HR = 0.93; 95% CI: 0.89-0.98, p = 0.004).

Treatment modality

**Figure 6:**
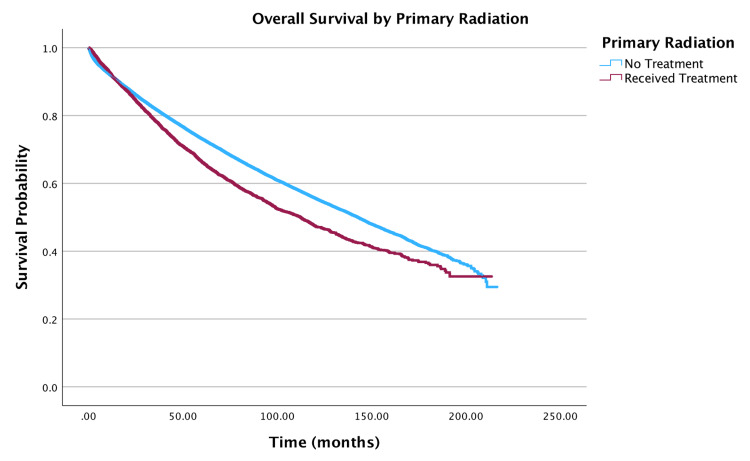
Overall survival by primary radiation treatment of patients with adenocarcinoma within adenomatous polyps (N = 58,643)

Treatment modality significantly impacted survival. Overall survival by primary radiation treatment of the tumor is shown in Figure 6. Primary radiation therapy was associated with a 28% higher hazard of death (HR = 1.28; 95% CI: 1.22-1.34, p < 0.001), while primary chemotherapy (HR = 0.86; 95% CI: 0.81-0.91, p < 0.001) and adjuvant chemotherapy (HR = 0.79; 95% CI: 0.74-0.83, p < 0.001) correlated with reduced hazards. Due to mutual exclusivity between neoadjuvant and adjuvant therapies in the dataset, the multivariable model accounted for neoadjuvant chemotherapy (encompassing both treatment sequences). A similar approach was applied to radiation therapy.

## Discussion

This study provides a thorough evaluation of demographic, clinical, and treatment variables influencing survival in patients with invasive tumors, offering insights into key determinants of outcomes. Our findings reveal that, in terms of the tumor itself, advanced stage, location in the sigmoid colon, distant metastasis, and primary radiation therapy are associated with worse prognosis, while treatment with primary and adjuvant chemotherapy is associated with increased survival probabilities. Related to patient demographic factors and socioeconomic status (SES), we found that increased age, male sex, Black race, lower education status, residing in low-SES neighborhoods, and non-private insurance are associated with lower survival probabilities.

Prior studies of a large cohort of CRC patients report a higher prevalence of early-onset tumors in the sigmoid colon and rectum, a finding consistent with the observed predominance of these sites in our analysis of colon adenocarcinoma [14,15]. In line with our findings of decreased survival in sigmoid colon and rectal tumors, previous research has documented more frequent adverse morphological features, perineural invasion, and venous invasion in both sigmoid and rectal cancers, suggesting a poorer prognosis for both locations [14].

Standard initial treatment for adenocarcinoma arising in adenomatous polyps typically involves polypectomy, performed under colonoscopic guidance to remove the lesion. However, the necessity for subsequent, more extensive surgical resection in the form of segmental colonic or rectal resection with lymphadenectomy is determined by specific pathological findings from the polypectomized specimen. These criteria include poor differentiation, lymphovascular invasion, and perineural invasion [16,17]. Depending on the stage of cancer, patients may also need adjuvant therapies (chemotherapy, radiotherapy, targeted therapy, and immunotherapy) [6,15]. Combined radiation and chemotherapy have been shown to significantly improve survival in Stages II and III rectal cancer [10]; additionally, adjuvant therapy is the standard of care for Stage III colon cancer and Stages II to III rectal cancer [6], a finding supported by our observation of significantly increased 5- and 10-year survival probabilities in patients receiving primary chemotherapy and adjuvant chemotherapy. Although our model identified sex as an independent predictor of overall survival, we did not explore potential interactions between sex and the receipt of chemotherapy or radiation. Future research could investigate whether the effectiveness of these treatments varies by sex in patients with adenocarcinoma within adenomatous polyps. Surgery is considered the primary treatment for early-stage CRC due to its effectiveness in eradicating local lesions [18]. Our data also indicated that cancer stage and treatment modality significantly influenced survival, with improved survival associated with adjuvant therapy and primary chemotherapy, and decreasing survival probabilities associated with increasing stage and primary radiation therapy. One potential explanation for this finding is confounding by indication. Radiation therapy is frequently indicated for locally advanced rectal cancers (late-stage) to improve local control and survival [19]. Patients with rectal tumors, particularly those with more advanced local disease, may inherently have a poorer prognosis compared to those with colon cancers or earlier-stage disease treated primarily with surgery. Our analysis, while adjusting for primary anatomic site and stage, might not have fully accounted for the nuances within these categories that led to the decision to administer primary radiation. Further research, with more detailed clinical data, including specific indications for radiation therapy, treatment regimens, and disease recurrence patterns, is necessary to clarify the role of radiation in this specific subtype of CRC and to disentangle the complex interplay of disease stage, location, treatment modality, and survival outcomes.

Limited literature exists specifically examining the association between socioeconomic factors and prognosis in adenocarcinoma arising in adenomatous polyps. However, studies employing multivariable analyses have investigated the relationship between various socioeconomic factors and overall CRC prognosis. Previous research has identified factors such as age, sex, and race as significant determinants of metastasis risk and overall prognosis in CRC [20]. Our study found that increasing age was associated with increased mortality risk and proportionately decreased 5- and 10-year survival estimates. The average age at diagnosis of CRC is 66 years [21], which matches our study, as the average age at diagnosis in our cohort was 66 years. The oldest age group (76-100 years old) had the lowest survival rates of 52% and 25% at 5 and 10 years, respectively. This aligns with findings from other studies demonstrating decreased survival with increasing age in patients with synchronous distant metastasis [6]. Overall, existing data demonstrate a higher incidence of CRC and lower survival rates in Black patients compared to other racial groups [6,22], consistent with our observation of higher mortality and lower survival in Black patients. Regarding sex, although males comprised the majority of our cohort (53%), females exhibited higher 5- and 10-year survival rates, consistent with previous reports of significantly higher overall five-year and disease-free survival (DFS) in women after curative resection [6].

Previous literature has shown an association between SES, assessed by individual education level or neighborhood-level measures, and CRC risk, even after adjusting for other risk factors. The overall incidence and mortality of CRC have been found to be significantly higher among individuals with low educational attainment [23,24], and individuals residing in low-SES neighborhoods have been found to have worse overall survival [25]. Our study similarly found decreasing 5- and 10-year survival probabilities in lower education level groups and in all lower income brackets. We also observed that insurance status is an important determinant of survival in colorectal adenocarcinoma, with patients on Medicare and Medicaid exhibiting decreased survival compared to those with private insurance. This is supported by previous findings that individuals with CRC who are uninsured or insured by Medicaid have higher mortality rates than those with commercial fee-for-service insurance [26]. Furthermore, previous research has identified insurance coverage as a critical predictor of CRC screening, which is associated with decreased mortality [10]. One proposed explanation for the observed survival disparities between Black and Caucasian races is differences in healthcare access and coverage [6,27].

The present study has inherent limitations. As a retrospective cohort study, it is subject to the limitations of this design, including reduced granularity compared to prospective studies. The retrospective nature of this study limits our ability to establish causal relationships between the identified factors and survival outcomes. The findings represent associations, and unmeasured confounders not captured within the NCDB may have influenced the observed results. Retrospective analyses, while valuable for exploring large datasets, are constrained by pre-existing data collection practices. Consequently, we could not capture granular clinical details that may influence outcomes, such as detailed treatment responses or comorbidities not recorded by the NCDB. Reliance on NCDB variables and the presence of missing data within our cohort further limited the scope of our analysis. While we attempted to adjust for potential confounders, the influence of unmeasured covariates on survival outcomes cannot be entirely excluded.

An additional limitation of our study is the designation of the "Other" racial group. The aggregation of non-White and non-Black racial/ethnic groups into an "Other" category was primarily due to the relatively smaller numbers within each of these groups in our NCDB dataset. We acknowledge that this group encompasses a heterogeneous mix of racial and ethnic populations, potentially masking distinct survival patterns within these subgroups. The interpretation of survival differences, when comparing "Other" race to White or Black races, should therefore be approached with caution.

A further limitation stems from the NCDB's recording of overall survival rather than cancer-specific survival (CSS), DFS, and recurrence-free survival (RFS). This precludes differentiation between deaths directly attributable to adenocarcinoma within adenomatous polyps and those due to other causes, particularly relevant in cohorts of older patients with competing risks for mortality. However, overall survival remains a relevant and meaningful endpoint, particularly in large-scale studies where CSS data may be unavailable. Given the relatively low incidence of adenocarcinoma arising specifically within adenomatous polyps, compared to all CRCs, overall survival provides a valuable metric for assessing long-term patient outcomes. Future research efforts, particularly those utilizing more granular clinical datasets or prospective studies, should prioritize the collection and analysis of DFS and RFS data to better understand the patterns of disease relapse and its contribution to poor outcomes in patients with adenocarcinoma within adenomatous polyps.

Despite these limitations, this study benefits from a relatively large sample size, particularly considering the specific focus on adenocarcinoma within adenomatous polyps. Utilizing the NCDB, which captures over 70% of new cancer diagnoses in the United States, strengthens the robustness and generalizability of our findings. However, the NCDB's exclusion of data from non-CoC-accredited facilities constitutes a potential limitation. This may introduce selection bias if there are systematic differences in patient populations or treatment patterns between CoC-accredited and non-accredited institutions. The magnitude of this potential bias is unknown.

## Conclusions

This study on adenocarcinoma within adenomatous polyps, involving 58,643 patients, aimed to identify key demographic and prognostic factors influencing survival. Our findings indicate that age, sex, race, and socioeconomic factors significantly influence survival outcomes, highlighting the need for targeted interventions to reduce disparities. Male sex and Black race had higher mortality risks compared to other gender and race groups. Medicare and Medicaid insurance were associated with increased mortality risk relative to private insurance. Older age, higher CD score, advanced NCDB cancer stage, and lower income were also linked to poorer outcomes. Early-onset tumors in the sigmoid colon and rectum had the worst prognosis. Distant metastasis, particularly involving the lymphatic system, significantly increased mortality risk across all cancer stages. These findings align with existing literature, though future studies should incorporate data from a broader range of facilities. The identification of older age, male sex, Black race, and lower SES as poor prognostic factors suggests the need for heightened awareness and potentially more aggressive monitoring or treatment strategies in these high-risk subgroups. Additionally, the significant impact of insurance status and socioeconomic factors underscores the importance of addressing barriers to healthcare access and quality for vulnerable populations with this diagnosis. Our study provides valuable insights into the demographics and prognostics of adenocarcinoma within adenomatous polyps through large-sample database analysis, allowing healthcare providers to enhance patient outcomes by caring for population subsets in a proactive and effective manner.
